# Mutations in DNA damage response pathways as a potential biomarker for immune checkpoint blockade efficacy: evidence from a seven-cancer immunotherapy cohort

**DOI:** 10.18632/aging.203670

**Published:** 2021-11-08

**Authors:** Wenjing Zhang, Liwen Zhang, Hao Jiang, Yuting Li, Suzhen Wang, Qinghua Wang

**Affiliations:** 1 Department of Health Statistics, Key Laboratory of Medicine and Health of Shandong Province, School of Public Health, Weifang Medical University, Weifang, Shandong 261053, China; 2 Tianjin Cancer Institute, National Clinical Research Center for Cancer, Key Laboratory of Cancer Prevention and Therapy of Tianjin, Tianjin Medical University Cancer Institute and Hospital, Tianjin 300060, China

**Keywords:** immunotherapy, DDR mutations, predictive biomarker, microenvironment, clinical practice

## Abstract

Recently several studies have demonstrated the implications of mutations in DNA damage response (DDR) pathways for immune checkpoint blockade (ICB) treatment. However, smaller sample sizes, lesser cancer types, and the lack of multivariate-adjusted analyses may produce unreliable results. From the Memorial Sloan-Kettering Cancer Center (MSKCC) cohort, we curated 1363 ICB-treated patients to evaluate the association of DDR mutations with immunotherapy prognosis. Besides, 4286 ICB-treated-naive patients from the Cancer Genome Atlas (TCGA) cohort were used to explore the intrinsic prognosis of DDR mutations. Factors in the microenvironment regarding DDR mutations were also assessed. We found that patients with DDR mutations exhibited a significantly prolonged immunotherapy overall survival via multivariate Cox model in the MSKCC cohort (HR: 0.70, *P* < 0.001). Specific cancer analyses revealed that patients with DDR mutations could obtain the better ICB prognosis in bladder cancer and colorectal cancer (HR: 0.59 [*P* = 0.034] and 0.33 [*P* = 0.006]). Stratified analyses showed that age >60, male gender, high mutation burden, and PD-1/PD-L1 treatment were the positive conditions for ICB survival benefits of DDR mutations (all *P* < 0.01). Mutations of 4 DDR genes, including *MRE11A*, *MSH2*, *ATM*, and *POLE* could predict favorable ICB prognoses (all *P* < 0.01). A better immune microenvironment was observed in DDR mutated patients. Mutations in DDR pathways or single DDR genes were associated with preferable ICB efficacy in specific cancers or subpopulations. Findings from our study would provide clues for tailing clinical trials and immunotherapy strategies.

## INTRODUCTION

Immune checkpoint blockade (ICB) therapies dramatically extended the survival interval of advanced tumors, however, the durable response was only observed in a subset of patients [1, 2]. Efficacy of ICB treatment could be predicted by multiple biomarkers, such as expression of programmed death receptor 1 ligand (PD-L1) [3, 4], tumor mutation burden (TMB) [5], neoantigen burden (NB) [6], mRNA expression signatures [7], and gut microbiome [8]. Their effectiveness would sometimes be lost in specific settings and each biomarker has a limiting application.

Difficulties in determining cut-off values, biases of distinct test platforms, and dynamic changes have reduced the broad utilization of PD-L1 expression [7]. Elevated TMB and NB were reported to be correlated with ICB treatment efficacy [5, 9], however, no uniform cut-off values were determined to select patients with a high mutational burden in distinct tumors [7]. Mismatch repair deficiency (dMMR) is another FDA-approved vital indicator owing to its ability to increase TMB and NB [1, 10]. However, less than 5% of tumor patients are dMMR-related, this reality may be a restrained factor for the extensive application of dMMR [11].

Six DNA damage response (DDR) signals (i.e., mismatch repair [MMR], nucleotide excision repair [NER], homologous recombination [HR], Fanconi anemia [FA], checkpoints, and specific DDR genes) are mainly existed to perform the function of genome maintenance, and thus preserve the genomic integrity [12]. Causally, alterations in any pathway or gene of DDR would induce the subtype with elevated TMB and MB [13]. Mutations of the MMR pathway were clinically correlated with durable ICB response [1, 14]. Mehnert et al. reported that endometrial cancer patients with *POLE* mutations exhibited favorable pembrolizumab efficacy [14]. Hugo et al. found that *BRCA2*-mutant melanoma patients harbored better clinical benefits of anti-PD-1 therapy [6]. Recently another study demonstrated that co-mutations of DDR signals were remarkably associated with elevated mutational load, increased immune signatures enrichment, better objective response rate, and prolonged ICB survival [15]. However, the limited sample size and cancer type of the above studies may influence the produced results. Besides, the predictive abilities of mutations of most DDR genes are poorly studied in clinical immunotherapy.

Herein, we curated a seven-cancer cohort to explore the association of DDR mutations with ICB efficacy in distinct subgroups. In addition, mutations of single DDR gene association with ICB clinical benefit were also evaluated. As a comparison, patients without ICB treatment from the Cancer Genome Atlas (TCGA) were used to assess the intrinsic prognostic ability of DDR mutations. Results from this study may give more implications for tailoring clinical ICB therapy.

## RESULTS

### Clinical characteristics and DDR mutations of included patients

Among 1363 ICB-treated tumor patients, 115 (8.4%) with BG, 211 (15.5%) with BLCA, 109 (8.0%) with CRC, 129 (9.5%) with HNSC, 344 (25.2%) with NSCLC, 142 (10.4%) with RCC, and 313 (23.0%) with SKCM. Overall, 1083 (79.5%) were treated with anti-PD-1/PD-L1 therapy, 76 (5.6%) were anti-CTLA-4 therapy, and 204 (14.9%) were combined therapy. The median ICB survival interval was 12 months. Other detailed clinical information was curated in Supplementary Table 1.

**Figure 1:**
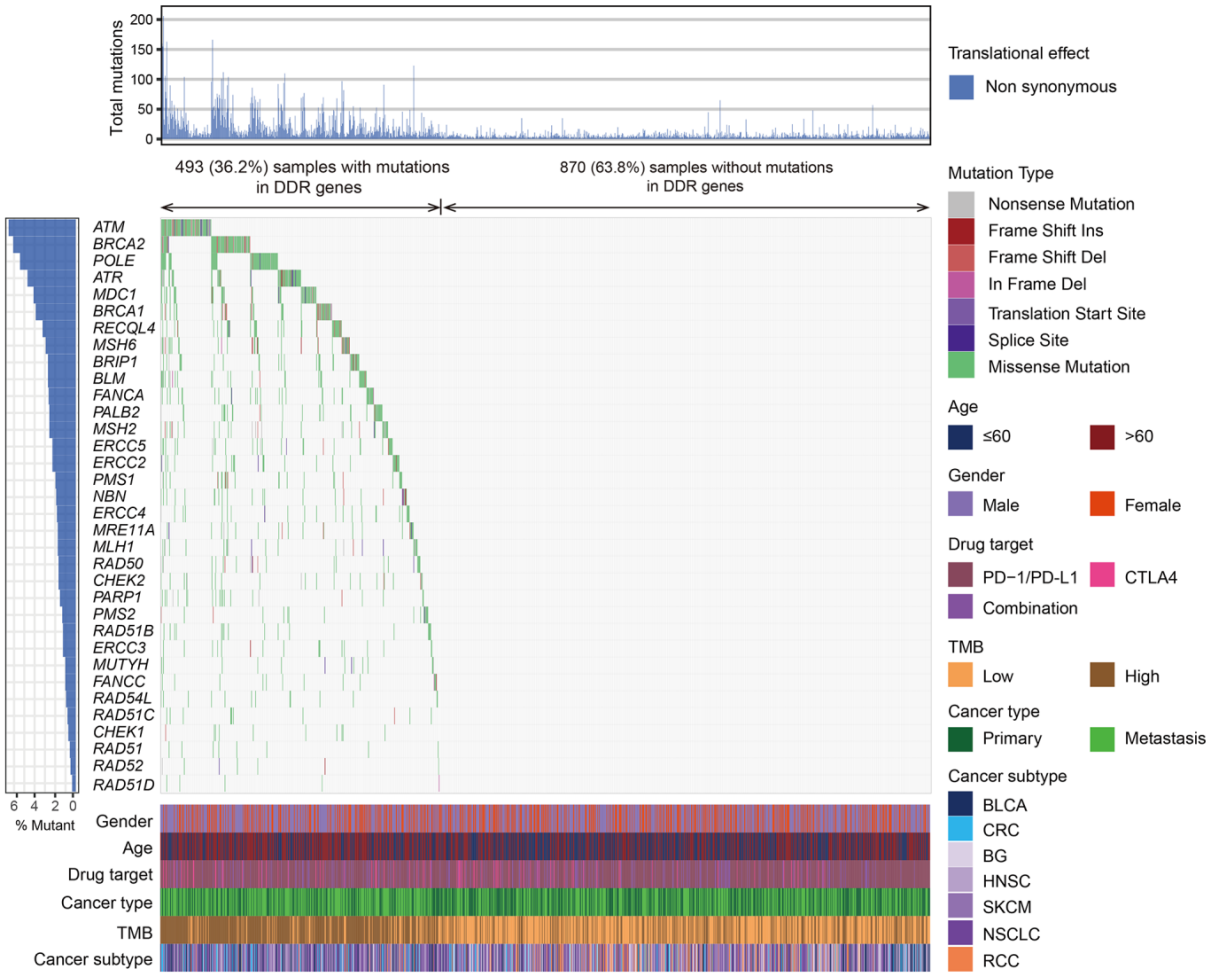
**The mutational pattern of 34 DDR genes among 1363 patients treated with ICB agents.** The left panel represents gene mutation rates, the upper panel indicates the non-synonymous mutation counts of each patient, the middle panel shows mutational landscape of all DDR genes with distinct mutation types color coded distinctly, and the bottom panel displays clinical characteristics such as age, gender, drug target, TMB, and cancer subtype.

Overall, 493 (36.2%) tumors harbored mutations in at least one DDR gene and 870 (63.8%) tumors were the DDR wild-type subgroup. The mutational patterns of 34 DDR genes were illustrated in Figure 1.

### Association of DDR mutations with prognosis in ICB-treated and ICB-treated-naive patients

**Figure 2:**
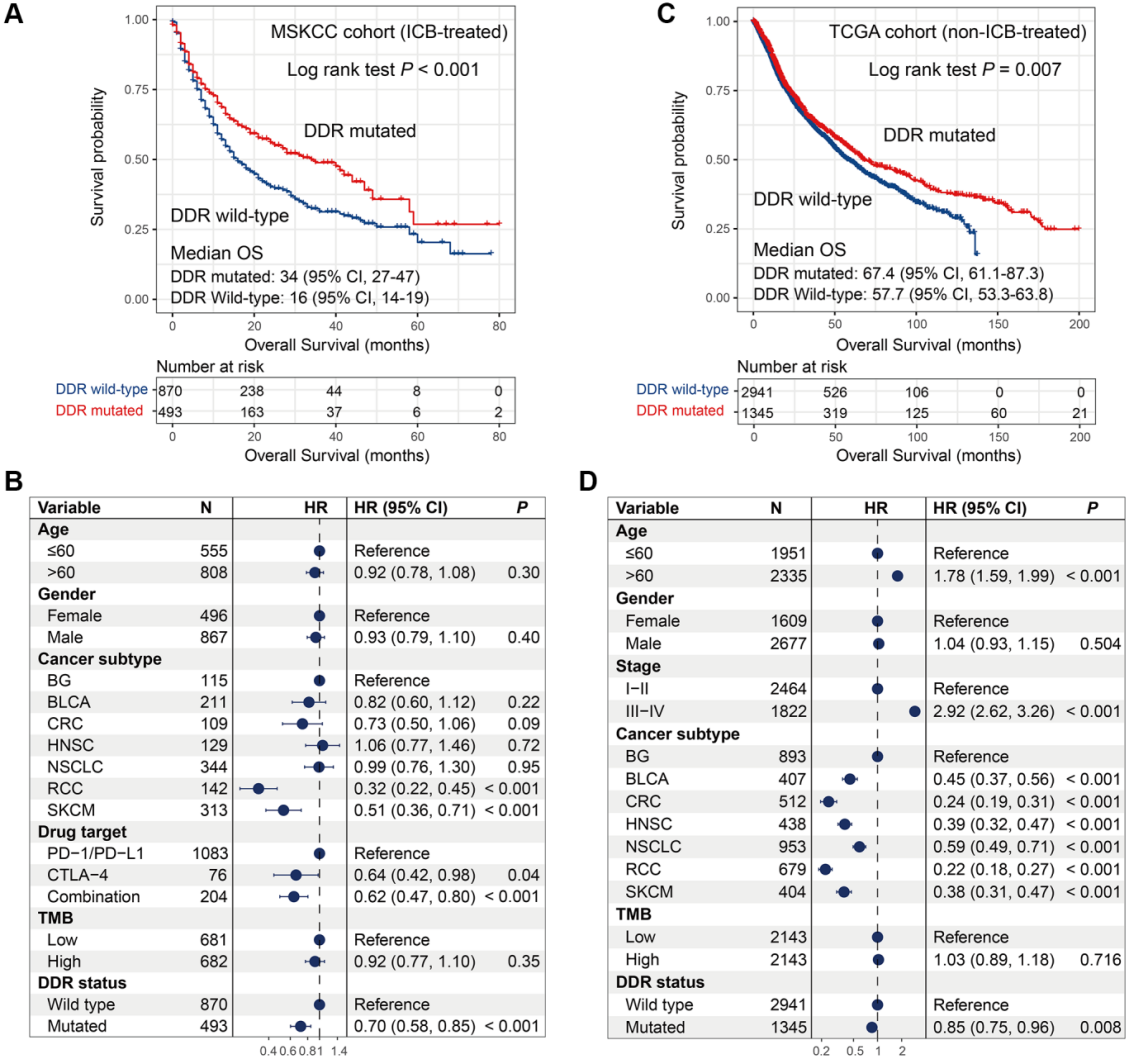
**Association of DDR mutations with survival outcome in MSKCC and TCGA cohorts.** (**A**–**B**) DDR mutations versus survival outcome with univariate analysis and multivariate regression model in the MSKCC cohort; (**C**–**D**) DDR mutations versus survival outcome with univariate analysis and multivariate regression model in the TCGA cohort.

In the MSKCC cohort contained ICB-treated patients, survival analysis showed that patients with DDR mutations exhibited a significantly better overall survival (OS) than patients without DDR mutations (median OS: 34 [95% CI, 27–47] vs. 16 [95% CI, 14–19] months; Log-rank test, *P* < 0.001; Figure 2A). To obtain a more accurate association, we conducted a multivariate Cox regression model with confounding variables (i.e., age, gender, cancer subtype, drug target, and TMB) taken into consideration. The result was still statistically significant (HR: 0.70, 95% CI: 0.58–0.85, *P* < 0.001; Figure 2B).

In the TCGA cohort, univariate survival analysis produced a consistent result as compared with the MSKCC cohort, that is DDR-mutated patients were significantly correlated with better prognosis (median OS: 67.4 [95% CI, 61.1–87.3] vs. 57.7 [95% CI, 53.3–63.8] months; Log-rank test, *P* = 0.007; Figure 2C). We also observed the significant association in multivariate Cox model after adjusting clinical confounders (HR: 0.85, 95% CI: 0.75–0.96, *P* = 0.008; Figure 2D).

DDR mutations were linked with favorable outcomes in both cohorts, however, DDR-mutated patients had more survival benefits in the MSKCC cohort than in the TCGA cohort (HR: 0.70 vs. 0.85; Figure 2B, 2D). These results indicate more positive roles of DDR mutations for predicting prognosis in immunotherapy settings.

### DDR mutations association with prognosis in 7 distinct cancers

We evaluated the prognostic ability of DDR mutations in 7 distinct cancer subtypes with MSKCC and TCGA cohorts. In this section, multivariate Cox models with confounders adjusted were performed only when survival curves were statistically significant.

**Figure 3:**
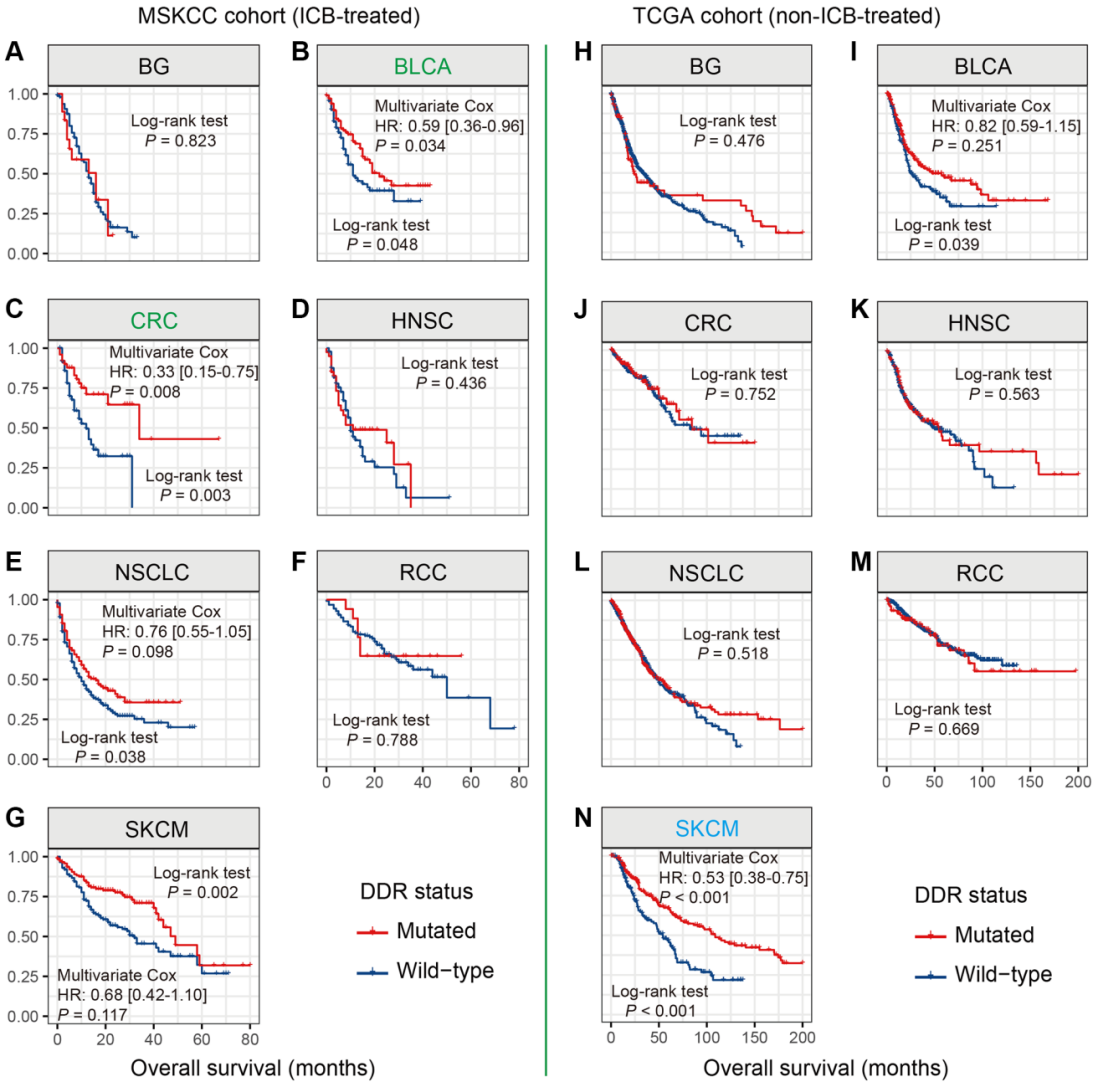
**DDR mutations association with prognosis in 7 distinct cancers.** (**A**–**G**) Association of DDR mutations with prognosis in 7 cancers in the MSKCC cohort; (**H**–**N**) Association of DDR mutations with prognosis in 7 cancers in the TCGA cohort. Cancer types that exhibited the survival benefits of *NLRP3* mutations in the MSKCC and TCGA cohorts were respectively colored with green and blue.

Univariate analysis in the MSKCC cohort showed that DDR mutations were significantly associated with favorable outcomes in 4 cancers, including BLCA, CRC, NSCLC, and SKCM (all log-rank test *P* < 0.05; Figure 3B, 3C, 3E, 3G). After controlling the confounding factors, only BLCA and CRC exhibited the survival benefits of DDR-mutated patients (HR: BLCA [0.59, 95% CI, 0.36–0.96, *P* = 0.034], CRC [0.33, 95% CI, 0.15–0.75, *P* = 0.008]; Figure 3B, 3C). DDR mutations in NSCLC and SKCM showed trends of better prognosis, however, they did not reach the statistical significance in multivariate analysis (HR: NSCLC [0.76, 95% CI, 0.55–1.05, *P* = 0.098], SKCM [0.68, 95% CI, 0.42–1.10, *P* = 0.117]; Figure 3E, 3G). We further used the TCGA data to calculate the intrinsic prognostic ability of DDR mutations in BLCA and CRC. Multivariate Cox model was not significant when controlling relevant confounders in BLCA (HR: 0.82, 95% CI: 0.59–1.15, *P* = 0.251; Figure 3I), and DDR-mutated CRC patients were also not related to the prognosis (Log-rank test *P* = 0.752; Figure 3J). The above results suggest the potential prediction implications of DDR mutations for immunotherapy efficacy in patients with BLCA and CRC.

SKCM patients with DDR mutations did not exhibit the survival benefits in the MSKCC cohort. However, in the non-ICB-treated TCGA cohort, DDR-mutated SKCM patients harbored a significantly better survival outcome than wild-type patients (HR: 0.53, 95% CI: 0.38–0.75, *P* < 0.001; Figure 3N).

DDR mutations were not correlated with patients’ survival in BG, HNSC, NSCLC, and RCC in both MSKCC and TCGA cohorts (all Log-rank test *P* > 0.05; Figure 3A, 3D, 3F, 3H, 3K, 3L, 3M).

### DDR mutations versus prognosis in distinct clinical conditions

To illuminate whether the immunotherapy and prognostic implications of DDR mutations were influenced by specific clinical factors, we performed stratified analysis and multivariate Cox regression model of DDR mutations in distinct subpopulations with MSKCC and TCGA data.

**Figure 4:**
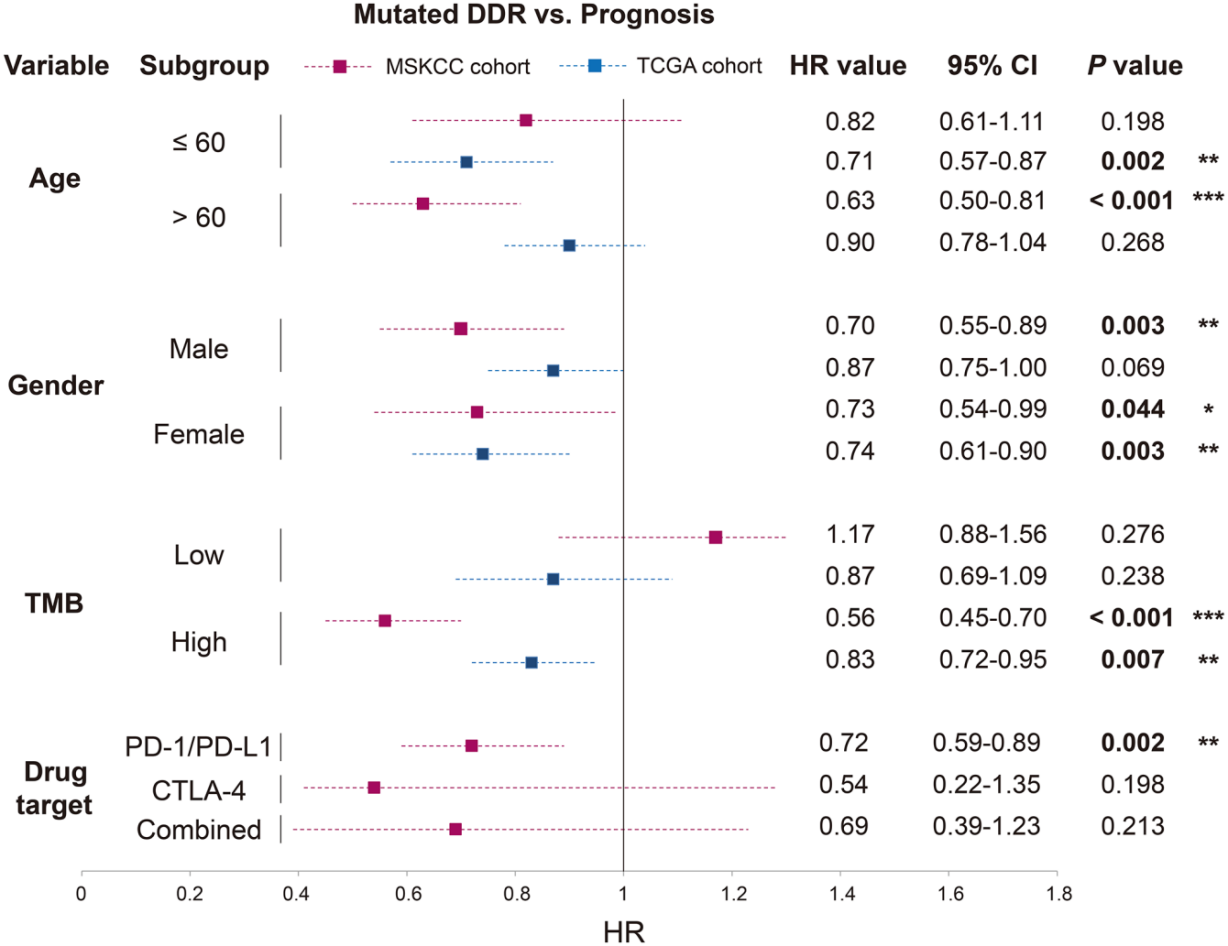
**Association of DDR mutations with survival outcome in distinct clinical settings based on the data from MSKCC and TCGA cohorts.** HR value, 95% CI, and *P* value were derived from multivariate Cox regression model with clinical factors adjusted. ^*^P < 0.05; ^**^P < 0.01; ^***^P < 0.001.

For patients with age ≤ 60, no significant correlation was observed between DDR mutations and prognosis in MSKCC (*P* = 0.198), however, DDR mutations could predict favorable survival in TCGA (*P* = 0.002). DDR-mutated patients with age > 60 harbored a better prognosis in MSKCC (*P* < 0.001), this result was not significant in TCGA (*P* = 0.268) (Figure 4; Supplementary Table 2).

Both male and female patients exhibited the survival benefits of DDR mutations in the ICB-treated cohort (*P* = 0.003 and 0.044). In the further analysis in TCGA, DDR-mutated male patients were not predictive of prognosis (*P* = 0.069), but female patients with DDR mutations obtained a more significant result (*P* = 0.003) (Figure 4; Supplementary Table 2).

DDR mutations could not predict prognosis in the low-TMB subgroup of both cohorts (both *P* > 0.05). However, patients with high TMB and DDR mutations harbored improved survival times in both cohorts (*P* < 0.001 and *P* = 0.007), and the result was more significant in the MSKCC cohort than in the TCGA cohort (HR: 0.56 vs. 0.83) (Figure 4; Supplementary Table 2).

Patients from MSKCC who received distinct immunotherapies also exhibited inconsistent association between DDR mutations and survival outcomes. DDR mutations were positively correlated with immunotherapy prognosis in patients treated with PD-1/ PD-L1 agents (*P* = 0.002), rather than patients treated with CTLA-4 agents or combined therapy (both *P* > 0.05) (Figure 4; Supplementary Table 2).

### Mutations of single DDR gene versus prognosis

To understand the implications of each DDR gene for immunotherapy efficacy and prognosis, we evaluated the association of mutations in single DDR genes with patients’ survival in 2 cohorts.

**Figure 5:**
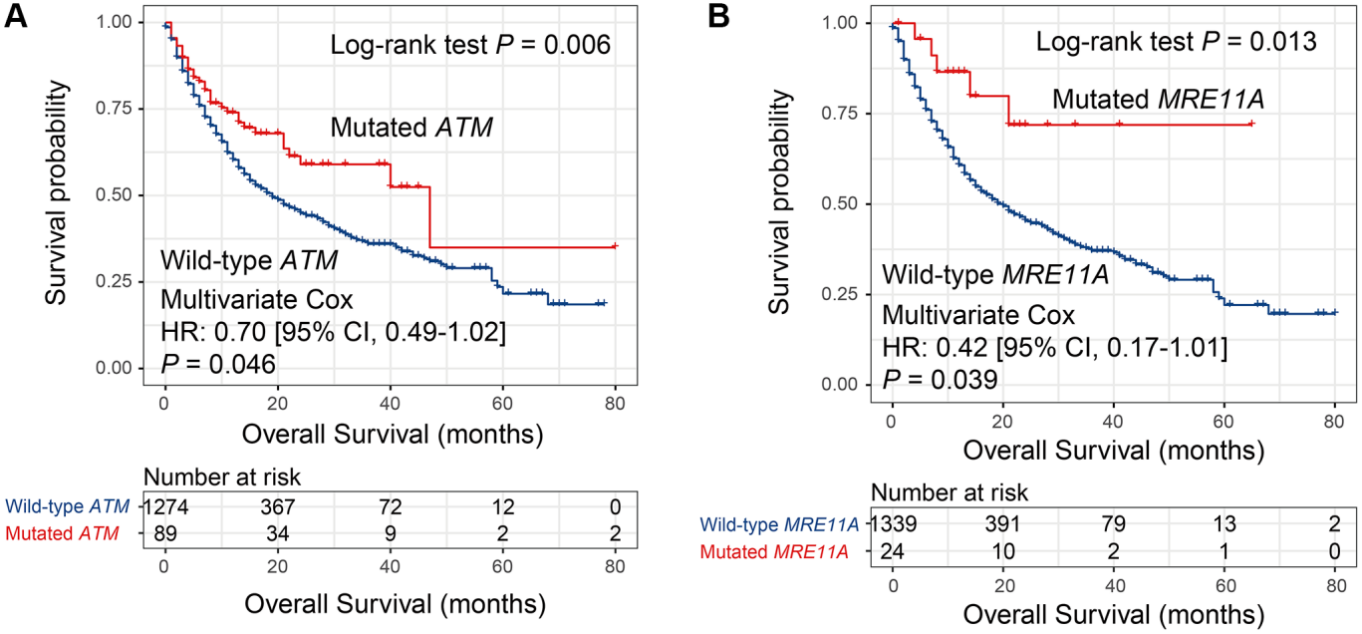
**Univariate and multivariate Cox regression analyses of mutations in 2 DDR genes in the MSKCC cohort.** Survival curves representation of mutations in (**A**) *ATM* and (**B**) *MRE11A*.

Univariate survival analysis in the MSKCC cohort showed that 8 DDR genes (i.e., *MSH2*, *MRE11A*, *NBN*, *BRCA2*, *RAD51C*, *ATM*, *POLE*, and *PARP1*) mutations were associated with the preferable survival outcome (all Log-rank *P* < 0.05; Supplementary Table 3). Multivariate Cox regression model was conducted with the mutations of identified 8 DDR genes and clinical confounding factors (i.e., age, gender, cancer type, drug target, and TMB) taken into consideration. Results showed that 2 genes, including *ATM* and *MRE11A*, still exhibited the positive association with prognosis (both multivariate-adjusted *P* < 0.05; Figure 5, Supplementary Figure 1). And these 2 genes mutations association with prognosis in TCGA were not statistically significant (both *P* > 0.05; Supplementary Table 3).

Besides, in the TCGA cohort, Kaplan-Meier survival analysis revealed that 3 DDR genes (i.e., *MSH6*, *BRCA1*, and *ATR*) mutations were related to the survival outcome (all Log-rank *P* < 0.05; Supplementary Figure 2). And this association remained still significant even adjusted for confounding variables (all multivariate-adjusted *P* < 0.05; Supplementary Figure 3). Mutations in *BRCA1* or *ART* were correlated with better survival outcomes, however, *MSH6* mutations were predictive of worse prognosis.

### Factors in the microenvironment concerning DDR mutations

We explored the correlation between microenvironment factors and DDR mutations to explain why DDR-mutated patients harbored better immunotherapy prognosis.

**Figure 6:**
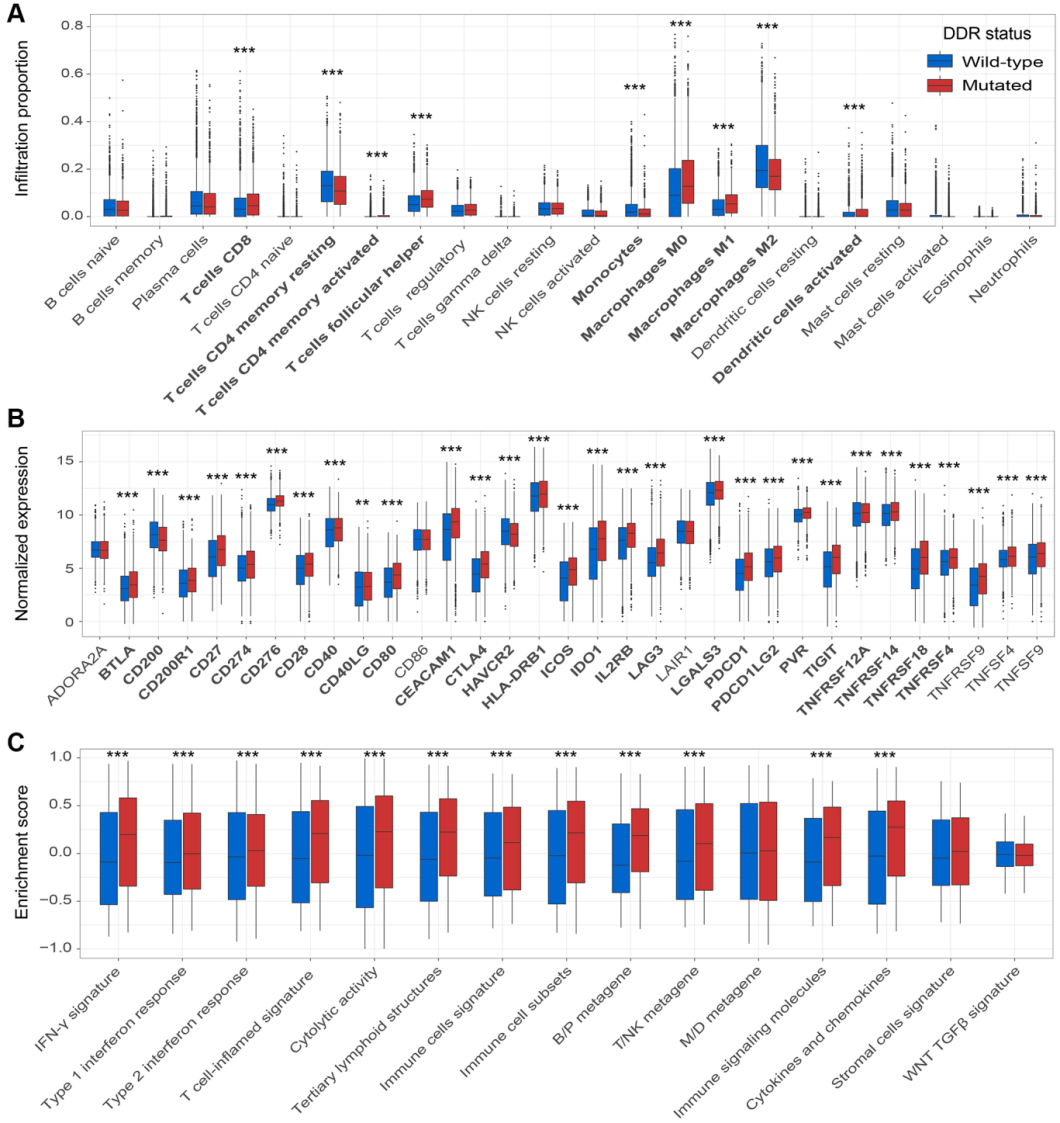
**DDR mutations association with factors in the immune microenvironment.** (**A**) Diverse infiltration abundance of 22 immune cells based on DDR mutational status; (**B**) Distinct expression of 33 immune checkpoints in patients with and without DDR mutations; (**C**) Distinct enrichment of 15 immune signatures in patients with and without DDR mutations. ^*^P < 0.05; ^**^P < 0.01; ^***^P < 0.001.

Tumor infiltration CD8 T cells were significantly enriched in the patients with DDR mutations (*P* < 0.001; Figure 6A). Macrophages M1 and M2 play immune-promotion and immune-suppressive roles, respectively. We observed that DDR-mutated patients had elevated infiltration of M1 macrophages and decreased infiltration of M2 macrophages (both *P* < 0.001; Figure 6A). Besides, patients with DDR mutations also harbored the elevated infiltration of resting and activated memory CD4 T cells, T follicular helper cells, monocytes, and activated dendritic cells (all *P* < 0.001; Figure 6A).

Among 33 immune checkpoints, 27 (81.8%) were significantly up-regulated in DDR-mutated patients, such as *CD274*, *PDCD1*, and *CTLA4* (all *P* < 0.001; Figure 6B). These results further verify the potential implications of DDR mutations for predicting ICB efficacy.

Of the 15 curated immune-related signatures, 12 (80.0%) were highly enriched in mutated DDR patients (all *P* < 0.001; Figure 6C). Especially, enrichment of IFN-γ signature and T cell-flamed signature, which were previously reported to be predictive of better ICB prognosis, were observed in DDR-mutated patients (both *P* < 0.001; Figure 6C).

## DISCUSSION

Previously several studies have reported the roles of DDR mutations in immunotherapy [12, 15]. However, smaller sample sizes, lesser cancer types, and lack of multivariate-adjusted analysis may introduce some biases into the generated results. In this study, by using an aggregated ICB cohort with 7 cancers, we performed multi-dimension analyses between DDR mutations and ICB treatment prognosis. As a comparison, non-ICB-treated patients from the TCGA cohort were also used to determine the potential ICB prediction and intrinsic prognosis abilities of DDR mutations. Several novel discoveries of our study would provide clues for tailing immunotherapeutic strategies.

Patients with DDR mutations exhibited more survival benefits in the MSKCC cohort as compared with the TCGA cohort. DDR pathways play vital roles in maintaining genome integrity; and mutations in DDR signals could produce the acceleration of genomic alterations (i.e., TMB). Previous many studies have demonstrated that a high TMB was linked with the preferable immunotherapy efficacy [16–20]. This may be a reason for explaining the more survival benefits of DDR mutated patients in the MSKCC. In addition, the increased infiltration of immune-response cells, the decreased infiltration of immune-suppressive cells, and elevated enrichment of numerous immune-related signatures were markedly enriched in patients with DDR mutations. These immune relevant factors to some extent contribute to the better prognosis of DDR mutated patients; however, they could play larger roles in the settings with immunotherapy. In a word, the more survival benefits of DDR mutations in the MSKCC cohort may be associated with the elevated TMB and favorable immune microenvironment.

DNA is continually exposed to the endogenous and exogenous damages, and the coordinated activity of multiple DDR pathways is needed to maintain genomic integrity under normal cellular conditions [21, 22]. Mutations in DDR signals could result in the failure to repair DNA damage and induce a variety of genomic alterations [10]. In some cases, changes produced by these genomic aberrations may serve as the antigens to the immune system and thus drive tumor initiation and immunogenicity [23, 24]. Acceleration of the genomic changes may be recognized as the neoantigens by the immune system, and push it to release more immune activity factors. The open microenvironment couples with immunotherapy could markedly enhance the treatment effects. In this study, DDR mutated patients harbored the preferable ICB survival outcomes, which may be implicated in the elevated TMB and neoantigen burden.

In specific cancer analysis, DDR mutations were correlated with favorable ICB survival in BLCA and CRC patients, while this correlation was not found in the other 5 cancers (i.e., BG, HNSC, NSCLC, RCC, and SKCM). Wang et al. used the integrated genomic and immunotherapy data and reported that the ICB treatment prognosis of patients with DDR mutations was superior to that of those without DDR mutations in NSCLC and SKCM [15]. The inconsistent results may attribute to the smaller sample size Wang et al. used as compared with our study (sample size: NSCLC (34 vs. 344), SKCM (174 vs. 313)). Although the significant association of DDR mutations with favorable ICB survival was not observed in SKCM patients received immunotherapy, in non-ICB-treated SKCM patients, DDR mutations were markedly correlated with better survival outcome, indicating that DDR mutations may play a more important role in predicting SKCM intrinsic prognosis rather than ICB prognosis. The firstly comprehensive analysis between DDR mutations and prognosis across multiple cancers demonstrated that BLCA or CRC patients with DDR mutations may obtain a prolonged survival interval in immunotherapy settings.

Previous studies have pointed out the age and sex differences in immune response and immunotherapy efficacy [25, 26]. In our study, younger (age ≤ 60) and female patients with DDR mutations did not exhibit the ICB treatment benefits. Inversely, older (age > 60) and male patients with DDR mutations harbored a remarkably better ICB prognosis. Consistent with our results, a recent study reported that younger and female patients always obtain a poorer response in clinical studies, this phenomenon may be correlated to the more poorly presented drive mutations these patients accumulated [27]. DDR-mutated patients with a high TMB also exhibit a favorable ICB prognosis. The high TMB, which is a stimulating factor for the activation of lymphocyte T cells, may provide a suitable environment for immunotherapy. Noticeably, in patients received anti-PD-1/PD-L1 agents, DDR mutations were linked with preferable survival outcome. However, this result was not observed in patients who received anti-CTLA-4 agents or combined therapy. By analyzing the association of DDR mutations with ICB efficacy in distinct clinical settings, we found that age > 60, male gender, high TMB, and anti-PD-1/PD-L1 treatment are the positive factors for the immunotherapy prognosis of DDR mutations.

Instead of choosing TMB or NB as predictors for immunotherapy efficacy, mutations in single genes, such as *POLE* [28], *POLD1* [28], *PBRM1* [29], *MUC16* [30, 31], and *TTN* [32] could also obtain the equivalent effects. In this study, mutations in 2 DDR genes (i.e., *ATM* and *MRE11A*) were associated with better ICB survival. Among, the positive link between *ATM* mutations and ICB benefits in BLCA was recently demonstrated [33], and this link was further verified based on 7 cancers in our study. Besides, *MRE11A* mutations correlation with improved survival was also firstly discovered in this study. These 2 genes we reported may harbor vital implications for evaluating immune checkpoint-based therapy efficacy.

We finally explored the links between factors in the microenvironment and DDR mutations. Immune cells represented by CD8 T cells infiltration, immune checkpoints (e.g., *CD274*, *PDCD1*, and *CTLA4*) expression, and immune-related signatures (e.g., IFN-γ and T cell-inflamed signatures) were highly enriched in the patients with DDR mutations. The better immune microenvironment may be the explanation for the preferable ICB prognosis of DDR mutations.

By using the aggregated ICB cohort and performing multi-dimension analyses, we found that DDR mutations were associated with better ICB survival outcomes in pan-seven-cancers. Besides, in specific cancers (e.g., BLCA and CRC) and in distinct clinical settings (e.g., age > 60, male gender, high TMB, and anti-PD-1/PD-L1 treatment), patients with DDR mutations also exhibited the ICB survival benefits. Mutations in *MSH2*, *MRE11A*, *ATM*, and *POLE* were all correlated with the favorable ICB prognosis. Findings derived from our study would provide evidence and basics for guiding clinical immunotherapy.

## MATERIALS AND METHODS

### Genomic data and clinical information of included patients

Somatic mutation data and clinical information of 1363 patients treated with ICB therapy were collected from the Memorial Sloan-Kettering Cancer Center (MSKCC) [16]. Among, brain glioma (BG), bladder cancer (BLCA), colorectal cancer (CRC), head and neck cancer (HNSC), non-small cell lung cancer (NSCLC), renal cell carcinoma (RCC), and melanoma (SKCM) were included for related analyses. Detailed clinical characteristics of these patients were curated in Supplementary Table 1.

Mutation and clinical data of 4286 patients contained above 7 cancers in TCGA were downloaded from Genome Data Commons (https://portal.gdc.cancer.gov). A total of 4021 patients with both gene expression and mutation data were used for immune microenvironment analysis.

### DDR genes and determination of DDR mutations

From a previous study reported by MSKCC [12], we collected 34 DDR-related genes of 6 pathways (Supplementary Table 4). Patients with nonsynonymous mutations (i.e., missense mutation, nonsense mutation, frameshift indel, inframe indel, splice site, and translation start site) of DDR genes were considered to be DDR-mutated.

### DDR mutations versus tumor-immune microenvironment

Based on the gene expression data from the TCGA cohort, we calculated and evaluated the enrichment of 3 factors in the microenvironment (i.e., tumor infiltration immune cells, immune checkpoint, and immune-related signatures) according to DDR mutation status.

Tumor infiltration immune cells proportion was calculated with the CIBERSORT algorithm, which is a useful tool to estimate the abundances of 22 immune cell types with gene expression data [34].

Ye et al. integrated a list of 34 immune checkpoint genes [35], in this study, the gene of *VSIR* was not found in the mRNA expression profile. Therefore, the expression of 33 immune checkpoints was analyzed.

Immune signatures that represented distinct immunological and cellular features were aggregated as follows: 1) Interferon-γ (IFN-γ) signature, which exhibits vital roles in activation and promotion of anti-tumor immune response, and it was reported to be associated with immunotherapy clinical benefits [36]; 2) T cell-inflamed signature, which is consisted of 18 immune genes correlated with dendritic and CD8 T cells activity [36]; 3) cytolytic activity, which reflects the activity of cytotoxic T cells and its released cytolytic factors to kill tumor cells [37]; 4) tertiary lymphoid structures (TLS), which are ectopic lymphoid organs related to cancer prognosis, immunity response, and ICB therapy efficacy [38]; 5) immune and stromal cells signature, which indicates the proportion of immune and stromal cells in mixed tumor tissue [39]; 6) immune cell subsets, which means the abundance of T cells, B cells, and natural killer (NK) cells [40]; 7) B/P, T/NK, and M/D metagenes were reported to be correlated with the enrichment of B cells/plasma cells, T cells/NK cells, and monocytes/dendritic cells, respectively [41]; 8) immune signaling molecules [40]; 9) cytokines and chemokines [40]; and 10) WNT TGFβ signature, which plays the suppression roles in immune response [42].

### Gene set variation and enrichment analysis

Enrichment scores of the abovementioned immune signatures were assessed with single sample gene set enrichment analysis (ssGSEA) method from GSVA package (V1.36.1) [43] according to the expression values of each gene in signatures. Differential analysis of TCGA sequencing data was performed with DESeq2 package (V1.28.1) [44]. Gene set enrichment analysis (GSEA) embedded in fgsea package (V1.14.0) (https://github.com/ctlab/fgsea) was utilized to calculate pathways enriched in distinct subgroups. Pathways from the Kyoto Encyclopedia of Genes and Genomes (KEGG) were used as the background dataset.

### Statistical analyses

R software (V4.0.1) and its packages were used to performed relevant analyses. The mutational pattern exhibited in the waterfall plot was drawn via GenVisR package (V1.20.0) [45]. Kaplan-Meier approach and Log-rank test were used to generate survival curves and to compare the difference significance of two curves, separately. Through forestmodel package (V0.5.0), multivariate Cox regression models were performed to adjust confounding factors and to produce forest plots. For the association of continuous variables with DDR mutation status, Wilcoxon rank-sum test was utilized. *P* values less than 0.05 were considered to be statistically significant unless a particular specification.

## Supplementary Materials

Supplementary Figures

Supplementary Table 1

Supplementary Tables 2-4
