# Supplementary material for: Mutations in DNA damage response pathways as a potential biomarker for immune checkpoint blockade efficacy: evidence from a seven-cancer immunotherapy cohort
Source: Aging (Albany NY). 2021 Nov 8;13(21):24136–54. doi: 10.18632/aging.203670 (PMC8610133; doi:10.18632/aging.203670)
Supplement: Supplementary Figures [file aging-13-203670-s001.pdf]

SUPPLEMENTARY FIGURES

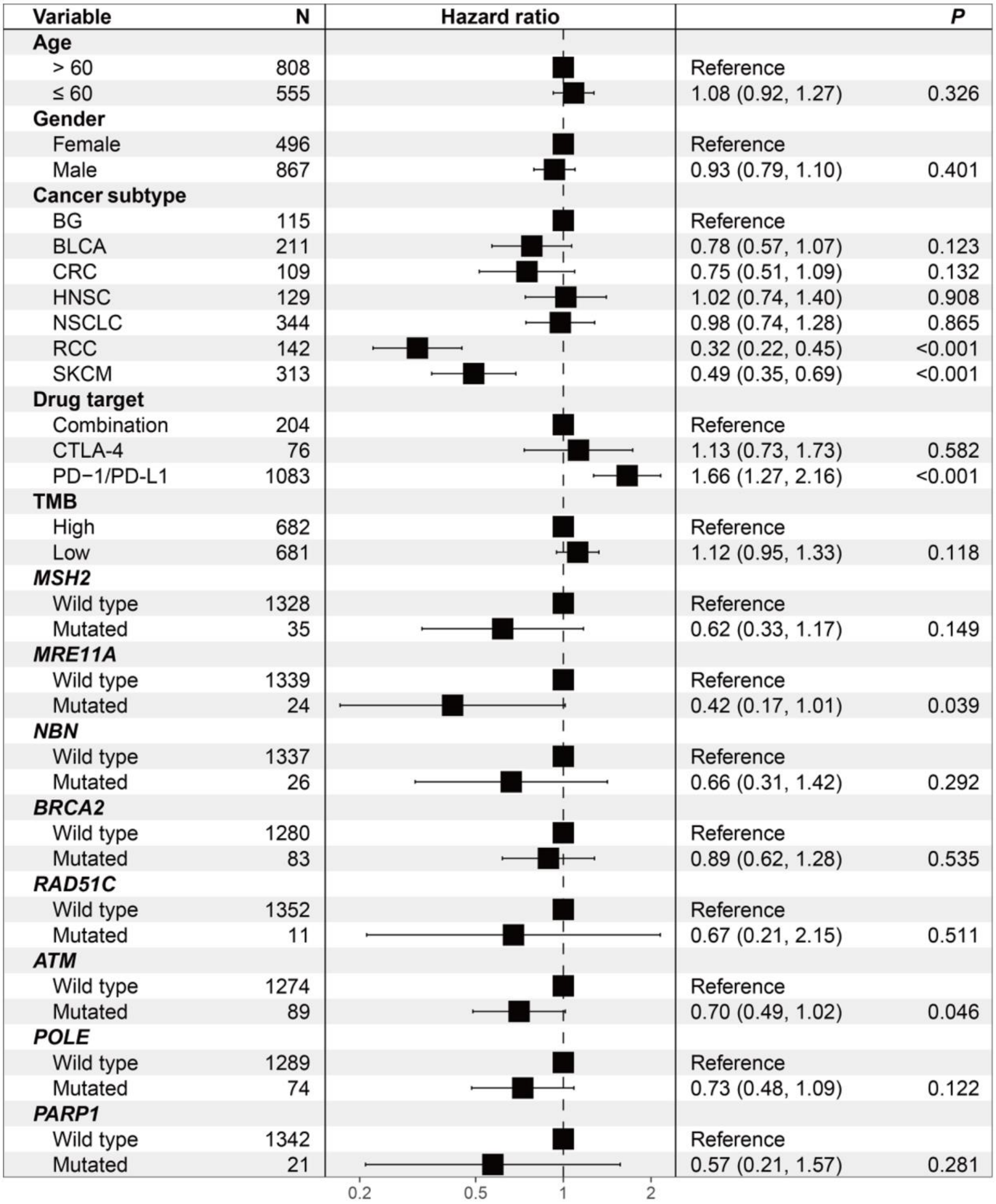

Supplementary Figure 1. Multivariate Cox regression model was conducted with clinical confounding factors and identified 8 DDR genes mutations taken into consideration in the MSKCC cohort.

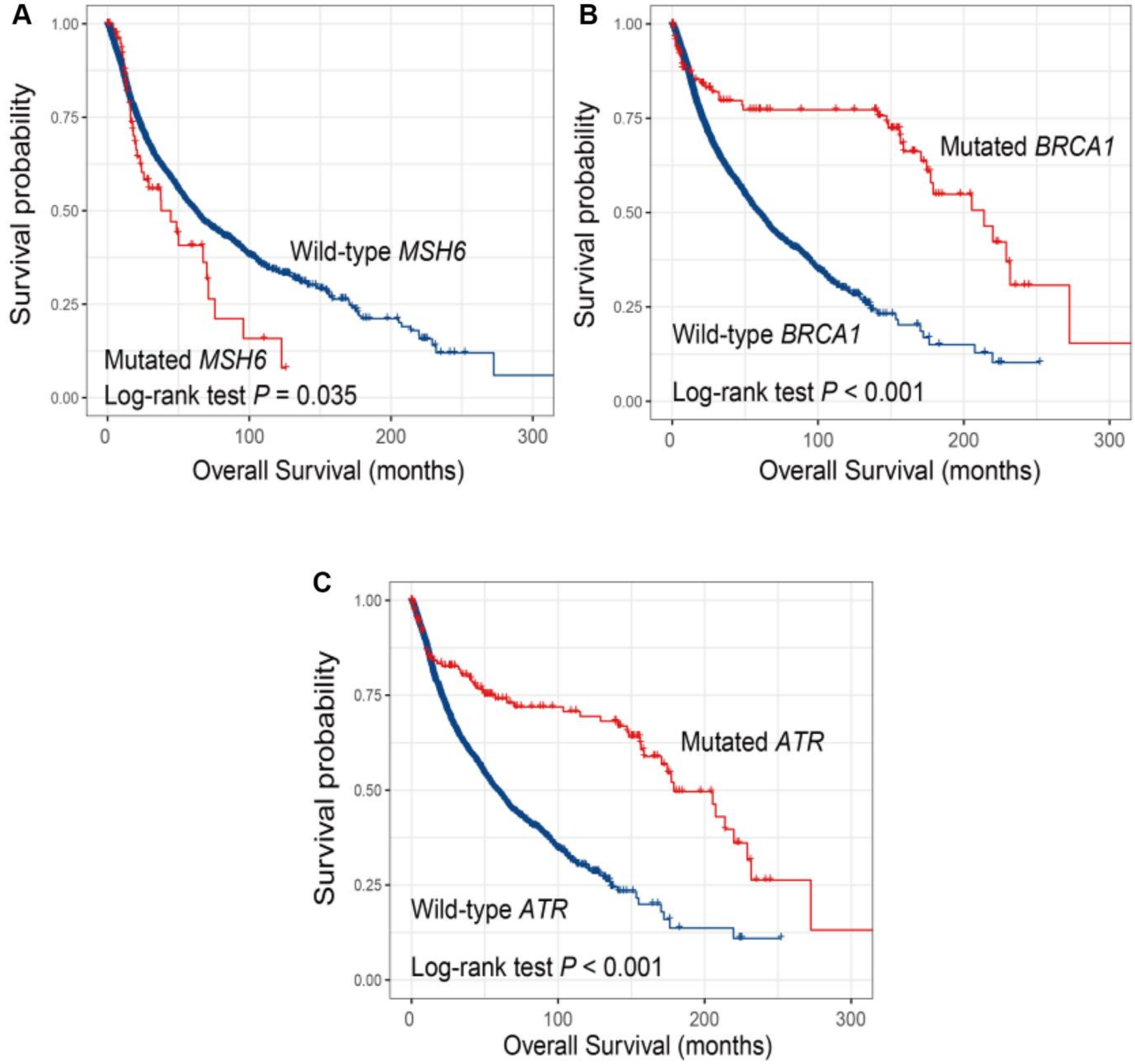

Supplementary Figure 2. Kaplan-Meier survival curves of mutations in 3 DDR genes in the TCGA cohort. Survival curves representation of mutations in (**A**) *MSH6*, (**B**) *BRCA1*, and (**C**) *ATR*.

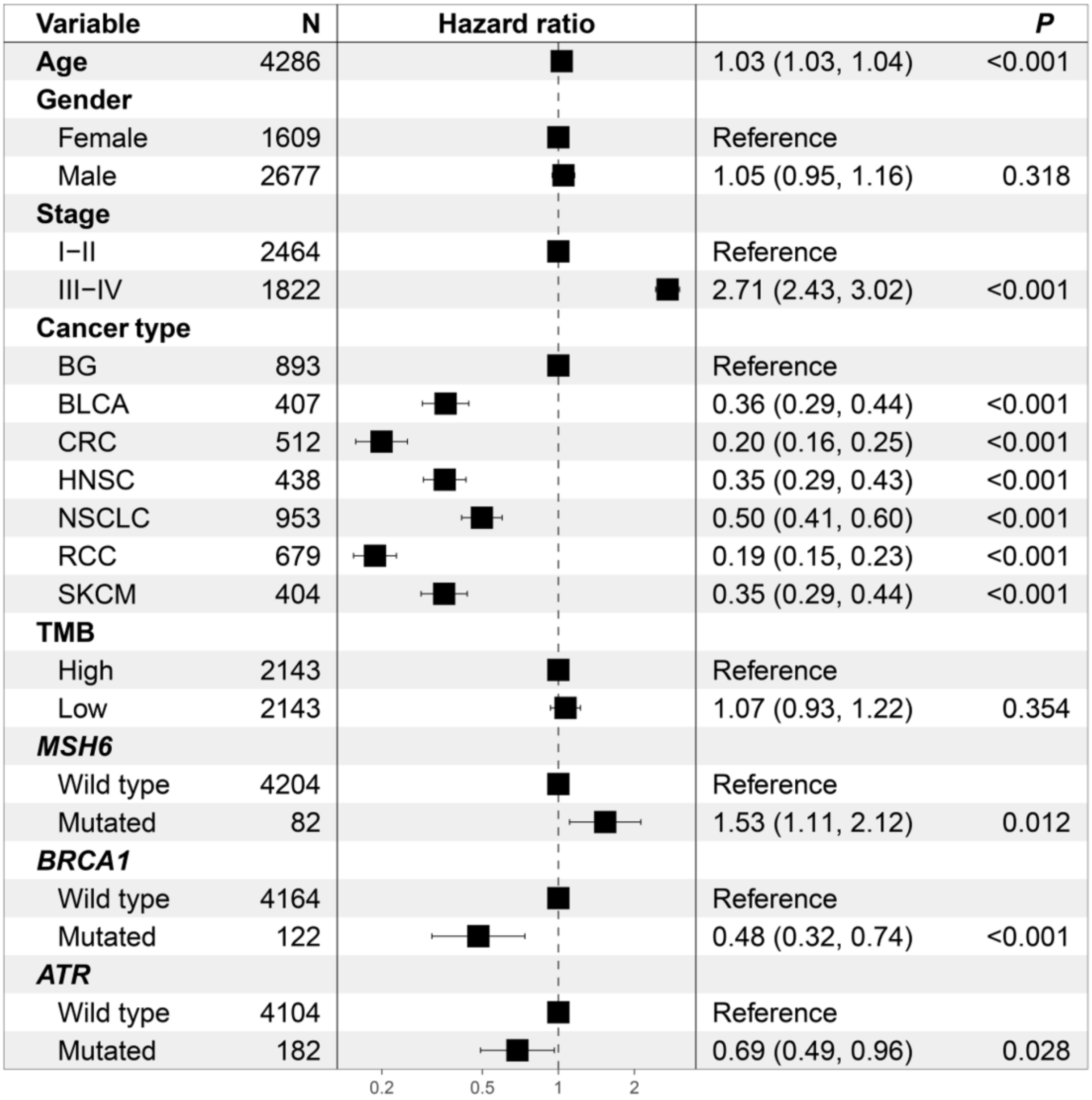

Supplementary Figure 3. Multivariate Cox regression model was conducted with clinical confounding factors and identified 3 DDR genes mutations taken into consideration in the TCGA cohort.
