# Supplementary material for: Mutations in DNA damage response pathways as a potential biomarker for immune checkpoint blockade efficacy: evidence from a seven-cancer immunotherapy cohort
Source: Aging (Albany NY). 2021 Nov 8;13(21):24136–54. doi: 10.18632/aging.203670 (PMC8610133; doi:10.18632/aging.203670)
Supplement: Supplementary Tables 2-4 [file aging-13-203670-s003.pdf]

SUPPLEMENTARY TABLES

Supplementary Table 2. Association of DDR mutations with prognosis in distinct conditions in 2 cohorts.

Variables	MSKCC cohort			TCGA cohort		
	HR*	95% CI	*P* value*	HR*	95% CI	*P* value*
**Age**						
≤60	0.82	0.61–1.11	0.198	0.71	0.57–0.87	**0.002**
>60	0.63	0.50–0.81	**<0.001**	0.9	0.78–1.04	0.268
**Gender**						
Male	0.7	0.55–0.89	**0.003**	0.87	0.75–1.00	0.069
Female	0.73	0.54–0.99	**0.044**	0.74	0.61–0.90	**0.003**
**TMB**						
Low	1.17	0.88–1.56	0.276	0.87	0.69–1.09	0.238
High	0.56	0.45–0.70	**<0.001**	0.83	0.72–0.95	**0.007**
**Drug target**						
PD-1/PD-L1	0.72	0.59–0.89	**0.002**			
CTLA-4	0.54	0.22–1.35	0.198			
Combined	0.69	0.39–1.23	0.213			

*HR and *P* Values were obtained via multivariate Cox model adjusted confounding factors.

Supplementary Table 3. Mutations of single DDR gene association with survival interval in 2 cohorts.

	MSKCC cohort	TCGA cohort
	Kaplan-Meier *P* value	Kaplan-Meier *P* value
MLH1	0.986	0.59
MSH2	0.027	0.091
MSH6	0.085	0.035
PMS1	0.872	0.213
PMS2	0.845	0.923
ERCC2	0.213	0.312
ERCC3	0.126	0.285
ERCC4	0.108	0.129
ERCC5	0.127	0.099
BRCA1	0.545	<0.001
MRE11A	0.013	0.413
NBN	0.028	0.716
RAD50	0.055	0.941
RAD51	0.224	0.644
RAD51B	0.078	0.645
RAD51D	0.412	0.535
RAD52	0.955	0.346
RAD54L	0.145	0.481
BRCA2	0.024	0.634

BRIP1	0.058	0.616
FANCA	0.064	0.382
FANCC	0.206	0.557
PALB2	0.368	0.945
RAD51C	0.035	0.654
BLM	0.493	0.846
ATM	0.006	0.358
ATR	0.188	< 0.001
CHEK1	0.456	0.756
CHEK2	0.635	0.978
MDC1	0.213	0.018
POLE	0.023	0.335
MUTYH	0.297	0.145
PARP1	0.017	0.923
RECQL4	0.334	0.112

Supplementary Table 4. DDR-related genes and pathways.

Genes + A2:B36	Pathways
MLH1	Mismatch repair (MMR)
MSH2	Mismatch repair (MMR)
MSH6	Mismatch repair (MMR)
PMS1	Mismatch repair (MMR)
PMS2	Mismatch repair (MMR)
ERCC2	Nucleotide excision repair (NER)
ERCC3	Nucleotide excision repair (NER)
ERCC4	Nucleotide excision repair (NER)
ERCC5	Nucleotide excision repair (NER)
BRCA1	Homologous recombination (HR)
MRE11A	Homologous recombination (HR)
NBN	Homologous recombination (HR)
RAD50	Homologous recombination (HR)
RAD51	Homologous recombination (HR)
RAD51B	Homologous recombination (HR)
RAD51D	Homologous recombination (HR)
RAD52	Homologous recombination (HR)
RAD54L	Homologous recombination (HR)
BRCA2	Fanconi anemia (FA)
BRIP1	Fanconi anemia (FA)
FANCA	Fanconi anemia (FA)
FANCC	Fanconi anemia (FA)
PALB2	Fanconi anemia (FA)
RAD51C	Fanconi anemia (FA)
BLM	Fanconi anemia (FA)

ATM	Checkpoint
ATR	Checkpoint
CHEK1	Checkpoint
CHEK2	Checkpoint
MDC1	Checkpoint
POLE	Others
MUTYH	Others
PARP1	Others
RECQL4	Others
